# Patients with Rheumatoid Arthritis Were Associated with a Risk of Rotator Cuff Diseases

**DOI:** 10.3390/jcm8020129

**Published:** 2019-01-22

**Authors:** Wei-Te Wang, Shih-Wei Huang, Tsan-Hon Liou, Hui-Wen Lin

**Affiliations:** 1 Department of Physical Medicine and Rehabilitation, Changhua Christian Hospital, Changhua 50006, Taiwan; 121330@cch.org.tw; 2 Department of Physical Medicine and Rehabilitation, School of Medicine, College of Medicine, Taipei Medical University, Taipei 11031, Taiwan; 13001@s.tmu.edu.tw (S.-W.H.); peter_liou@s.tmu.edu.tw (T.-H.L.); 3 Graduate Institute of Sports Science, National Taiwan Sport University, Taoyuan 33301, Taiwan; 4 Department of Physical Medicine and Rehabilitation, Shuang Ho Hospital, Taipei Medical University, New Taipei City 23561, Taiwan; 5 Department of Mathematics, Soochow University, Taipei 11102, Taiwan; 6 Evidence-Based Medicine Center, Wan Fang Hospital, Taipei Medical University, Taipei 11696, Taiwan

**Keywords:** rheumatoid arthritis, rotator cuff disease, risk factor, population-based study

## Abstract

Rheumatoid arthritis (RA) commonly causes inflammation in the joints and periarticular structures. The association between RA and rotator cuff (RC) has been reported; however, epidemiological studies on RA and RC tendons are scant. Therefore, we investigated RC disease (RCD) risk and analyzed the effects of RA medication, steroids, and methotrexate, on the risk of RCD for patients with RA. We conducted a retrospective cohort study with a 6-year longitudinal follow-up in Taiwan. Patients who received RA diagnoses between 2004 and 2008 were enrolled in the study cohort. The non-RA control cohort comprised age- and sex-matched controls. Propensity score matching was used for other comorbidities and treatments. The hazard ratios (HRs) and adjusted HRs (aHRs) were estimated after confounders were adjusted for. Effects of steroid and methotrexate use on RCD risk were also analyzed. We enrolled 4521 RA patients (study cohort) and 22,605 matched controls. RCD incidence was 145 and 91 per 100,000 person-years in the RA and control cohorts, respectively. In the RA cohort, the crude HR for RCD was 1.62 (95% confidence interval (CI), 1.41–1.86, *p* < 0.001), and the aHR was 1.56 (95% CI, 1.36–1.79, *p* < 0.001). The methotrexate nonusers exhibited an aHR (vs. controls) of 1.61 (95% CI, 1.40–1.85, *p* < 0.001), but the methotrexate users did not have a significantly higher aHR than the controls. The steroid nonusers had an aHR (vs. controls) of 1.69 (95% CI, 1.46–1.96, *p* < 0.001), but the aHR of the steroid users was not significantly higher than the control aHR. Patients with RA had a higher risk for RCD compared with the non-RA control cohort. Steroids or methotrexate use significantly reduces the risk of RCD occurrence in patients with RA. Treatment for RCD symptoms and controlling inflammatory process are important to ensure high-quality care for patients with RA.

## 1. Introduction

Rheumatoid arthritis (RA) is an autoimmune disease characterized by chronic inflammation, which can cause bone and cartilage destruction in the joints [1]. Patients with RA usually exhibit symptoms of painful symmetrical joint swelling, warmth, and morning stiffness [2]. These symptoms of RA can interfere with daily activities and adversely affect quality of life. In Asia, RA has a mean onset age of 50 years, exhibits female predominance, and affects 0.1%–0.3% of the general population [3]. In Taiwan, the average age-adjusted annual incidence rate was 15.8 per 100,000 persons [4]. while the adjusted incidence rates were very stable at 20.9–25.2 per 100,000/year and 7.0–8.2 per 100,000/year for females and males, respectively. Besides the adjusted average incidence ratio of females/males was 3.1 [5]. In addition to affecting the synovial membrane that lines the joints, RA can affect tendon sheaths [6]. RA is characterized by hypervascularized synovitis, which causes bony erosion, damage of cartilage, destruction of joints, bone marrow edema, and bursitis [7]. Based on murine models, in the early stages of RA, tendon involvement could be the initial sign of inflammatory arthritis [8]. In addition, flexor tendon sheath involvement was considered an early indicator of RA [9]. Moreover, early assessment of tendon inflammation was considered crucial for the prevention of irreversible injuries with serious subsequent complications, such as tendon and joint damage [10]. Controlling the inflammation of extra-articular tissue, such as the tendon sheath near the synovial membrane, should be given special attention to improve the quality of care for patients with RA.

The rotator cuff (RC) consists of supraspinatus, infraspinatus, teres minor, and subscapularis muscles and tendons. These structures facilitate shoulder motion and provide dynamic stabilization to the glenohumeral joint. Injury to the musculoskeletal structures is known as RC disease (RCD). The risk factors of RCD can be classified into intrinsic factors such as overuse, chronic impingement syndrome, and extrinsic factors such as degeneration, and hypoperfusion [11]. Besides, a population based study also mentioned that diabetes mellitus (DM) and hyperlipidemia are independent risk factors for developing RCD [12]. Shoulder pain and functional disability are common symptoms in patients with RCD. RCD has an age-related prevalence, which was 2.8% in individuals aged >30 years and 15% in individuals aged >70 years [13]. RCD presents as an intrinsic age-related degenerative process. RCD covers multiple types of shoulder problems, such as tendonitis, impingement syndrome, RC tears, and subacromial bursitis. Patients with RCD often report shoulder pain while performing actions such as overhead throwing exercises or daily activities. A related study indicated that 30%–70% of patients with RCD presented with shoulder pain and the incidence of RC tears was 5%–40% [14]. Severe RCDs, such as RC tear or rupture, can result in a limited range of motion and dysfunction. The prevention of and effective treatment for RCD for maintaining a relatively high quality of life and the continuation of daily activities and work are crucial issues in public health.

In patients with RA, the subcutaneous extensor and flexor tendons of the hand are frequently ruptured and require surgical repair [15]. A recent cross-sectional study investigating hand tendons revealed the predominance of tenosynovitis or tendonitis in patients with RA [16]. Although RA often invades the tendons of the hand, to our knowledge there have been no large scaled epidemiological studies investigating the association between RA and RCD of the shoulder. For an improved understanding of the risk of RCD among patients with RA, we conducted this longitudinal retrospective case-control cohort study. Furthermore, in order to identify the pathogenesis mechanism, we analyzed the effects of RA medication such as steroids and methotrexate, on the risk of RCD for patients with RA.

## 2. Methods

### 2.1. Study Data Source, and Ethics Approval

The data of this study were obtained from the Longitudinal Health Insurance Database (LHID2005), which was provided by National Health Research Institutes (NHRI) in Taiwan. For research purposes, The Taiwan NHRI contains registration files and original claims data for reimbursement, which were derived from the National Health Insurance (NHI) administration. The LHID2005 contains the claims data of 1,000,000 beneficiaries randomly sampled from all the participants, who were registered for NHI coverage since 2005. The NHI program was launched in Taiwan in March 1995 and covers 99.9% of the Taiwanese population (approximately 23 million enrollees in 2012). It covers almost all medical services, such as outpatient visits, admission service, and emergency hospitalization, and provides information about diagnostic codes, demographic data (such as age and sex), treatments of medical prescription, procedures, and surgery. To protect the privacy of the patients, all the names and identification number data of the LHID2005 were replaced by codes comprising numbers and English letters. The informed consent requirement was waived because the patients could not be identified. This study was approved by the Institutional Review Board of the University of Taipei. (UT-IRB No.: IRB-2018-07).

### 2.2. Study Population Selection Process

**Figure 1:**
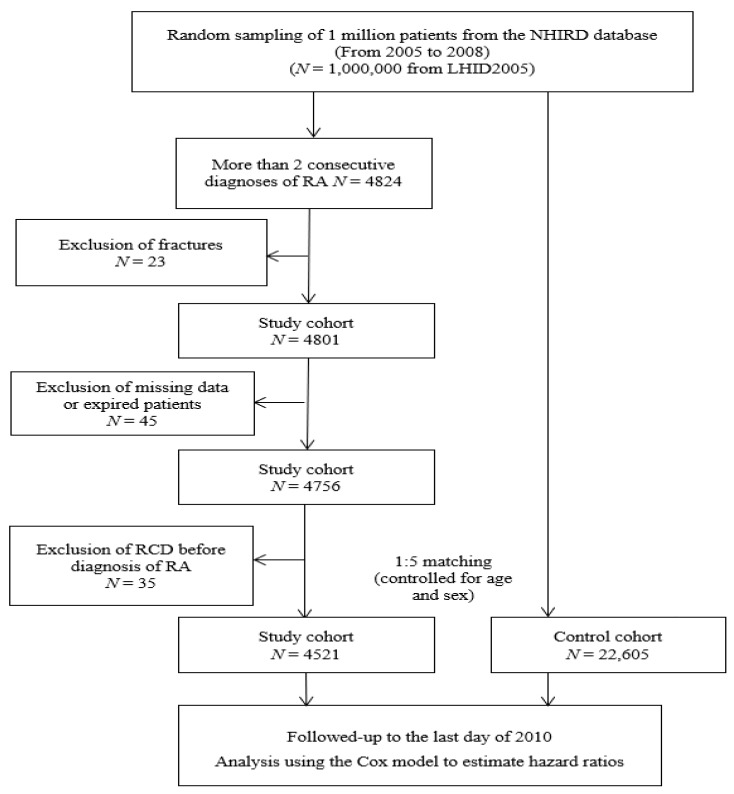
Flowchart showing study design.

In the LHID2005, the diseases were recorded using codes from the International Classification of Disease, Ninth Revision, Clinical Modification (ICD-9-CM) in the insurance claims data. The flowchart of the study population selection is presented in Figure 1. From the database, we selected an RA cohort aged >20 years based on the ICD-9-CM code 714.0 at diagnosis between 2005 and 2008. Patients who met at least four of the diagnostic criteria based on the 1987 American College of Rheumatology criteria were considered as having RA and those diagnosed by rheumatologists were included in the RA cohort [17]. To overcome concerns regarding the accuracy and possibility of incorrect coding, we only selected the patients with at least two instances of primary consecutive diagnosis of RA codes. Furthermore, when patients receive diagnoses of RA, they can apply for catastrophic illness certification in the NHI program and patients can receive waivers of copayments for RA-related medical expenditures. When patients apply for catastrophic illness certification, the NHI administration assigns a rheumatologist to review the application primarily based on American College of Rheumatology criteria for RA. RA patients with a previous diagnosis of RCD, (before 2004), with missing data, and who died during the follow-up period were excluded from this study. Finally, 4521 patients with RA were selected in the study cohort. In the control cohort, we matched the variables of sex and age at a 5:1 ratio with the study cohort.

### 2.3. Demographic Variables and Comorbidities

In LHID2005, the demographic variables of age, sex, and urbanization level, in addition to data on medications used and steroids or nonsteroidal anti-inflammatory drug use, were obtained for analysis. The comorbidities were diabetes mellitus (ICD-9-CM codes 250 and 251), hypertension (ICD-9-CM codes 401–405), coronary heart disease (ICD-9-CM code xx), thyroid disorders (ICD-9-CM codes 240–246), gout (ICD-9-CM code 274), and systemic lupus erythematosus (SLE) (ICD-9-CM code 710.0). To minimize the influence of bias from data selection from the study database, we used propensity scores that were adjusted for comorbidities and income, as shown in Table 1.

### 2.4. Outcome Determination

We used RCD (ICD-9-CM diagnostic codes 726.1: RC syndrome of the shoulder and allied disorders; 727.61: complete rupture of the RC; or 840.4: sprains and strains of the RC and capsule) as the endpoint from the index date, or 31 December 2010, whichever was earlier, and the final-date observations were censored observations.

### 2.5. Statistical Analysis

Demographic characteristics and comorbidities were analyzed using Pearson’s chi-square test. We calculated the incidence of RA and compared the risk of RCD between the two cohorts by using the Cox model after propensity score adjustment. Furthermore, we compared the risk of RCD in the patients with RA who did (users) or did not (nonusers) receive medication (methotrexate and steroids) for more than 20 days with the risk of RCD in the control cohort. To clarify the association between medication use and RCD, Kaplan-Meier hazard curves of RCD were plotted for methotrexate users or nonusers over a 6-year follow-up period. Similarly, steroid use was compared to examine the effects of steroid use on the risk of RCD among patients with RA. All data analyses were performed using the Stata package (Version 11) and SAS statistical package (Version 9.1.3; SAS Institute, Cary, NC, USA). A *p* value of < 0.05 was considered statistically significant.

## 3. Results

### 3.1. Demographic Characteristics and Comorbidities

Women constituted 71.7% of the RA (methotrexate users, methotrexate nonusers, steroid users, and steroid nonusers) and control cohorts. The prevalence of the comorbidities of hyperlipidemia, coronary heart disease, SLE, gout, and thyroid disorders were higher in the RA cohort than in the control cohort before propensity score matching was performed (Table 1).

### 3.2. Risk of RCD among RA Cohort

The incidence of RCD was 145 and 91 per 100,000 person-years in the RA and control cohorts, respectively. In the study cohort, the crude hazard ratio (HR) of RCD was 1.62 (95% CI, 1.41–1.86, *p* < 0.001), while the adjusted HR (aHR) was 1.56 (95% CI, 1.36–1.79, *p* < 0.001) (Table 2).

**Figure 2:**
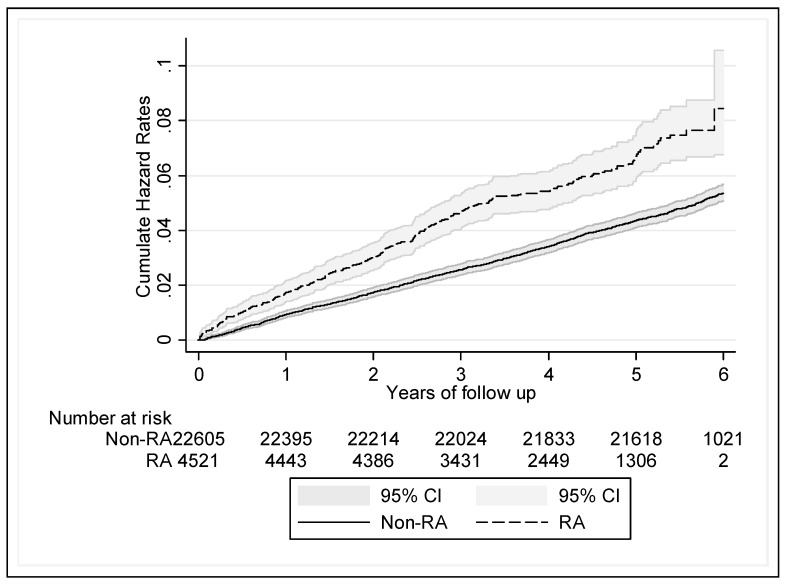
Kaplan-Meier hazard curve for rotator cuff (RC) disease (RCD) in patients with rheumatoid arthritis (RA) and those without RA for a (up to) 6-year follow-up (2005–2010).

Figure 2 reveals the Kaplan-Meier hazard curves for the risk of RCD in the RA cohort, and controls during the 6-year follow-up period. And the RA cohort revealed statistical significant higher cumulative HR than non-RA cohort.

### 3.3. Risk of RCD for RA Cohort with or without Methotrexate

The propensity score aHR of multivariable Cox regression for RCD in the methotrexate nonusers in the RA cohort was 1.61 (95% CI, 1.40–1.85, *p* < 0.001) and the aHR in the methotrexate users was not statistically different from that of the control cohort (Table 3).

**Figure 3:**
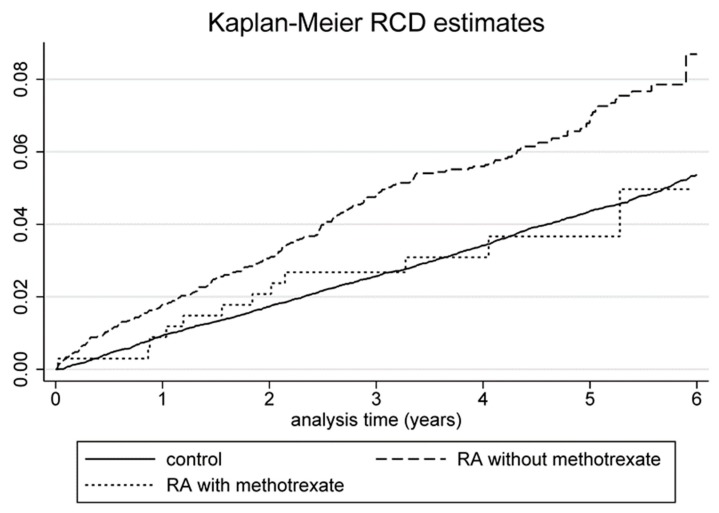
Kaplan-Meier hazard curve for RCD in RA patients with or without methotrexate use and controls over a 6-year follow-up.

Figure 3 depicts the Kaplan-Meier hazard curves for the risk of RCD in the RA cohort among methotrexate users and methotrexate nonusers, and controls during the 6-year follow-up period. The RCD risk was higher in those who did not use methotrexate.

### 3.4. Risk of RCD for RA Cohort with or without Steroid

Those who did not use steroids had a significantly higher risk of requiring RC repair surgery than the controls (aHR = 1.69, 95% CI, 1.46–1.96, *p* < 0.001), whereas the risk of RC repair surgery did not differ significantly between steroid users and the controls (Table 4).

**Figure 4:**
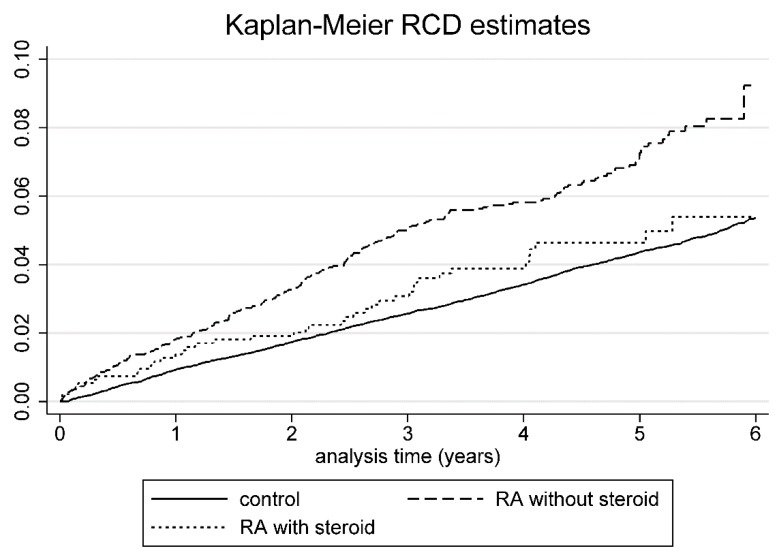
Kaplan-Meier hazard curve for RCD in RA patients with or without steroid use and controls over a 6-year follow-up.

Figure 4. shows the Kaplan-Meier hazard curves of RCD risk in the steroid users, steroid nonusers, and controls during the 6-year follow-up period. The risk of RCD increased among the steroid nonusers during the 6-year follow-up.

## 4. Discussion

Our longitudinal retrospective cohort study demonstrated that patients with RA were at higher risk of being diagnosed with RCD. This epidemiological study indicated that in addition to the joints of the hands and shoulders, attention should also be paid to the RC lesions. When patients with RA used steroids, the incidence of RCD was not significantly different from the control cohort. Our study findings were consistent with those of a previous cross-sectional study in which shoulder ultrasound examinations were conducted and 49% of the tested patients with RA exhibited RC pathology [18]. Studies have shown that the simultaneous involvement of small and large joints was uncommon among patients with RA, and the involvement of large joints was associated with a relatively high disease severity and disability [18,19]. Studies have also reported that the shoulder is commonly affected by RA; this limits the physical functions of patients with RA [20,21]. Our study finding indicates that during the 6-year longitudinal follow-up period, the number of events of RCD increased with RA progression. Identifying key pathogenesis mechanisms and developing effective RCD prevention strategies are necessary to improve the quality of life and prevent the negative effects of RCD in patients with RA. Our study also demonstrated that both methotrexate and steroids seem to aid in preventing RCD in patients with RA.

With regard to the possible etiologies of RCD, factors related to RC injuries can be classified into intrinsic and extrinsic factors [11]. In the patients with RA, we considered that intrinsic pathogenic etiologies played a crucial role in the occurrence of RCD. Patients with RA exhibit chronic systemic inflammation, which can invade the synovial membranes of multiple joints [22]. When chronic inflammation processes of RA involve the shoulder, the articular surface can be affected in addition to the glenohumeral joint, which can result in an RC tear [23]. Before an RC tear occurs, subclinical inflammation can occur and affect the related soft tissues of the shoulder, such as the bursa or labrium. Chronic inflammation can impair the remodeling and healing of the tendon and increase the vulnerability of patients with RA to subsequent RCD lesions. Patients with RA often present with proliferative synovitis, and it can affect adjacent structures, thus cause thickening of the bursa wall and tenosynovium [24]. Inflammation was considered a key pathogenesis factor of RCD in patients with RA.

Patients with RA frequently present with chronic inflammation. To control the progression of inflammatory lesions, systemic steroids are often used in combination with nonsteroidal medication, as they control flare-up episodes. Steroid use can accelerate weakness progression by inhibiting collagen synthesis and impairing blood supply [25]. A critical zone near the insertion of the supraspinatus has been described using microangiographic evidence of an area of hypovascularity in the tendon near its humeral insertion. Relative ischemia in this zone is reported to mimic tendon degeneration [14]. Corticosteroids inhibit collagen synthesis and may impair blood supply, thus weakening the tendons [26]. Hence, the vulnerability of RA patients to RC injuries and subsequent RCD increases. However, our study revealed that steroid use in all the RA patients and controls did not significantly increase the risk of RCD. We posited that steroid administration could arrest inflammation, although steroid use causes tendon degeneration. We hypothesized that although steroid use increases the risk of RC tear, it reduces the risk of RCD incidence among patients with RA; therefore, the overall effect of steroid use in terms of RCD risk would not differ significantly from the control cohort.

Our study showed that the methotrexate nonusers were at higher risk of receiving a diagnosis of RCD than the control cohort. However, there was no statistical difference of RCD risk among the methotrexate users and the control cohort. Methotrexate is one of the most commonly used disease-modifying antirheumatic drugs for patients with RA, and was considered a standard medication for RA. However, some patients cannot tolerate the adverse effects of methotrexate, such as hepatotoxicity and bone marrow suppression [27,28]. Methotrexate has an anti-inflammatory effect, and a related study revealed that it can reduce the incidence of vascular diseases [29]. In addition to its anti-inflammatory effect, methotrexate has an antithrombotic effect because it enhances protein functions required for the efflux of cellular cholesterols [30]. We supposed that the anti-inflammatory and antithrombotic effects of methotrexate could reduce RCD risk in patients with RA; RCD can be caused by chronic inflammation and impaired circulation of the tendon.

Our study revealed that patients with RA were at higher risk of RCD. We hypothesized that the possible mechanism underlying this association was chronic inflammation and tendon degeneration, which causes weakening of the RC and more vulnerability to injuries. The strength of this study was its large sample size. It is the first epidemiological study to investigate the risk of RCD among steroid or methotrexate users. However, this study has several limitations that should be addressed. First, the diagnoses of RA and comorbidities were based on the ICD codes from the database; hence, accuracy should be examined. To ensure accurate payment, the Bureau of NHI reviews medical records regularly. Patients with RA in Taiwan can apply for catastrophic illness registration cards, and the copayment is waived for RA-related medical problems by the Bureau. Furthermore, only the patients with at least two consecutive RA diagnoses were included in the study cohort to reduce the chances of incorrect coding. Second, the severity and disease activities of the patients with RA could not be classified because laboratory data cannot be obtained from the database. Besides, the influence of other RA related medication such as DMARDs, NSAIDs, and biologic therapies, which also affect the diseases activities were not analyzed in this study. However, some kinds of DMARDs, NSAIDs, and biologic therapy coding of pharmacy were not available nor adequate for statistical analysis. In order to investigate the risk of RCD and possible pathogenesis explanation, we decided to analyze the effects of RCD risk among RA patients by methotrexate and steroid. Third, extrinsic factors affecting RC injuries, including repeated impingement and overuse during work and daily living activities, cannot be analyzed using this database. Although a large sample size was obtained from this database, the effects of the aforementioned confounders cannot be excluded completely from this study. Finally, the severity, classification, definition of RCD, and affected side were not described in detail, and the study population was not stratified by site and severity in this study. Despite the scant information available on the types of RCDs, our population-based study showed that patients with RA were at risk of being diagnosed with RCD, and steroid or methotrexate use can reduce the risk of RCD to a low level that is comparable with the RCD risk in the control cohort.

## 5. Conclusions

This is the first large-scale cohort study to investigate the risk of RCD among patients with RA. Patients with RA were at a higher risk of being diagnosed with RCD than the control cohort. Further analysis indicated no statistical difference between the RCD risk in the patients with RA who used anti-inflammatory medications, such as steroids or methotrexate, and the control cohort. This implies that inflammation could be among the crucial factors contributing to RCD among patients with RA. Additional studies are recommended to clarify the correlation between the severity of RA and risk of RC tendon lesion to improving the quality of care for patients with RA. Moreover, this study finding could be applied to lower the risk of RCD among other kinds of inflammatory autoimmune diseases and further studies are required in the future.

## Figures and Tables

**Table 1:** Demographic characteristics and comorbidities for patients in the rheumatoid arthritis and control cohorts from 2004 to 2008.

Baseline Variable	RA Cohort *n* = 4521		Control Cohort *n* = 22605		*p* Value	After Propensity Scores Were Adjusted
	No.	(%)	No.	(%)		*p* Value
Characteristics						
Sex						
Female	3241	71.7	6400	71.7		
Male	1280	28.3	16205	28.3		
Age (years)						
18–30	318	7.0	1590	7.0		
31–40	535	11.8	2675	11.8		
41–50	981	21.7	4905	21.7		
51–60	1124	24.9	5620	24.9		
61–70	777	17.2	3885	17.2		
>70	786	17.4	3930	17.4		
Urbanization level					0.015	0.531
Urban	2527	55.9	13139	58.1		
Suburban	1386	30.7	6670	29.5		
Rural	608	13.4	2796	12.4		
Comorbid medical disorders						
DM					0.053	0.999
Yes	671	14.8	3108	13.7		
No	3850	85.2	19497	86.3		
Hypertension					0.308	0.467
Yes	1407	31.1	6862	30.4		
No	3114	68.9	15743	69.6		
Hyperlipidemia					<0.001	0.284
Yes	974	21.5	3763	16.6		
No	3547	78.5	18842	83.4		
Coronary heart disease					<0.001	0.725
Yes	629	13.9	2659	11.8		
No	3892	86.1	19946	88.2		
SLE					<0.001	0.753
Yes	54	1.2	45	0.2		
No	4467	98.8	22560	99.8		
Thyroid					<0.001	0.441
Yes	253	5.6	897	4.0		
No	4268	94.4	21708	96.0		
Stroke					0.122	0.877
Yes	354	7.8	1622	7.2		
No	4167	92.2	20983	92.8		
Gout					<0.001	0.061
Yes	748	16.5	1742	7.7		
No	3773	83.5	20863	92.3		

Abbreviations: DM: diabetes mellitus, SLE: systemic lupus erythematosus, RA: rheumatoid arthritis, RCD: rotator cuff disease.

**Table 2:** Crude and adjusted hazard ratios for rotator cuff disease (RCD) in the rheumatoid arthritis and control cohorts during the (up to) 6-year follow-up period, starting from the index date of an ambulatory care visit (*n* = 27,126).

Presence of RCD	Control Patients	Patients with RA
Up to 6-year follow-up period		
Yes/Total (%)	1171/22,605 (5.2%)	264/4521 (5.8%)
Person-years	128,638	18,254
Incidence per 10,000 person-years	91	145
Crude HR (95% CI)	1.00	1.62 * (1.41–1.86)
Propensity score adjusted HR (95% CI)	1.00	1.56 * (1.36–1.79)

Notes: Propensity scores were matched for age, sex, hypertension, stroke, hyperlipidemia and diabetes, coronary heart disease, SLE, thyroid, gout, and urbanization level. * indicates *p* < 0.001. Abbreviations: HR: hazard ratio, CI: confidence interval, SLE: systemic lupus erythematosus, RA: rheumatoid arthritis, RCD: rotator cuff disease.

**Table 3:** Incidence, crude and adjusted hazard ratios and 95% confidence intervals (CIs) for RCD during the (up to) 6-year follow-up. (*n* = 27,126).

Presence of RCD	Non-RA	Patients with RA	
	*n* = 22,605	RA without Methotrexate	RA with Methotrexate
		(*n* = 4184)	(*n* = 337)
Follow-up period			
Yes (%)	1171 (5%)	252 (6%)	12 (4%)
Incidence per 10,000 person-years	91	149	89
Crude HR (95% CI)	1.00	1.67 * (1.45–1.92)	1.00 (0.56–1.76)
Propensity score adjusted (95% CI)	1.00	1.61 * (1.40–1.85)	0.95 (0.53–1.68)

Notes: Propensity scores were made for age, sex, hypertension, stroke, hyperlipidemia and diabetes, coronary heart disease, SLE, thyroid, gout, and urbanization level. * indicates *p* < 0.001. Abbreviations: HR: hazard ratio, CI: confidence interval, SLE: systemic lupus erythematosus, RA: rheumatoid arthritis, RCD: rotator cuff disease.

**Table 4:** Incidence, crude and adjusted hazard ratios and 95% confidence intervals for RCD during the (up to) 6-year follow-up. (*n* = 27,126).

Presence of RCD	Controls	Patients with RA	
	(*n* = 22,605)	RA without Steroid (*n* = 3581)	RA with Steroid (*n* = 940)
Follow-up period			
Yes (%)	1171 (5%)	224 (6%)	40 (4%)
Incidence per 10,000 person-years	91	155	103
Crude HR (95% CI)	1.00	1.75 * (1.51–2.02)	1.15 (0.84–1.58)
Propensity score adjusted (95% CI)	1.00	1.69 * (1.46–1.96)	1.10 (0.80–1.51)

Notes: Propensity scores were made for age, sex, hypertension, stroke, hyperlipidemia and diabetes, coronary heart disease, SLE, thyroid, gout, and urbanization level. * indicates *p* < 0.001. Abbreviations: HR: hazard ratio, CI: confidence interval, SLE: systemic lupus erythematosus, RA: rheumatoid arthritis, RCD: rotator cuff disease.
